# Structure–Effect Relationships of Novel Semi-Synthetic Cannabinoid Derivatives

**DOI:** 10.3389/fphar.2019.01284

**Published:** 2019-11-20

**Authors:** Marcus R. Götz, Juan A. Collado, Javier Fernández-Ruiz, Bernd L. Fiebich, Laura García-Toscano, María Gómez-Cañas, Oskar Koch, Andreas Leha, Eduardo Muñoz, Carmen Navarrete, Maria R. Pazos, Ulrike Holzgrabe

**Affiliations:** ^1^Institute of Pharmacy and Food Chemistry, Julius-Maximilians-Universität Würzburg, Würzburg, Germany; ^2^Symbiosis Laboratories, Symrise AG, Holzminden, Germany; ^3^Vivacell Biotechnology España, Córdoba, Spain; ^4^Departamento de Biología Celular, Fisiología e Inmunología, Instituto Maimónides de Investigación Biomédica de Córdoba, Hospital Universitario Reina Sofía, Universidad de Córdoba, Córdoba, Spain; ^5^Departamento de Bioquímica y Biología Molecular, Facultad de Medicina, Instituto Universitario de Investigación en Neuroquímica, CIBERNED and IRYCIS, Universidad Complutense, Madrid, Spain; ^6^VivaCell Biotechnology GmbH, Denzlingen, Germany; ^7^Medical Biometry and Statistical Bioinformatics, Department of Medical Statistics, Georg-August-University of Göttingen, Göttingen, Germany

**Keywords:** cannabidiol, cannabidivarin, CBD, CBDV, CB-receptor, agonist, binding, anti-inflammatory

## Abstract

**Background:** As a library of cannabinoid (CB) derivatives with (-)-*trans*-cannabidiol (CBD) or (-)-*trans*-cannabidivarin (CBDV) scaffold, we synthesized nine novel cannabinoids: 2-hydroxyethyl cannabidiolate (2-HEC), 2-hydroxypentyl cannabidiolate (2-HPC), 2,3-dihydroxypropyl cannabidiolate (GCBD), cyclohexyl cannabidiolate (CHC), *n*-hexyl-cannabidiolate (HC), 2-(methylsulfonamido)ethyl cannabidiolate (NMSC), 2-hydroxyethyl cannabidivarinolate (2-HECBDV), cyclohexyl cannabidivarinolate (CHCBDV), and *n*-hexyl cannabidivarinolate (HCBDV). Their binding and intrinsic effects at the CB1- and CB2-receptors and the effects on inflammatory signaling cascades were investigated in in vitro and ex vivo cell models.

**Materials and Methods:** Binding affinity was studied in membranes isolated from CB-receptor-transfected HEK293EBNA cells, intrinsic functional activity in Chinese hamster ovary (CHO) cells, and activation of nuclear factor κB (NF-κB) and nuclear factor of activated T-cells (NFAT) in phorbol 12-myristate 13-acetate (PMA)/ionomycin (IO)-treated Jurkat T-cells. Inhibition of interleukin (IL)-17-induced pro-inflammatory cytokines and chemokines [IL-6, IL-1β, CC-chemokine ligand 2 (CCL2), and tumor necrosis factor (TNF)-α] was studied in RAW264.7 macrophages at the RNA level. Pro-inflammatory cytokine (IL-1β, IL-6, IL-8, and TNF-α) expression and prostaglandin E2 (PGE_2_) expression were investigated at the protein level in lipopolysaccharide (LPS)-treated primary human monocytes.

**Results:** Derivatives with long aliphatic side chains at the ester position at R^1^ [HC (**5**)] as well as the ones with polar side chains [2-HECBDV (**7**), NMSC (**6**), and 2-HEC (**1**)] can be selective for CB2-receptors. The CBDV-derivatives HCBDV and CHCBDV demonstrated specific binding at CB1- and CB2-receptors at nanomolar concentrations. 2-HEC, 2-HPC, GCBD, and NMSC were agonists at CB2-receptor and antagonists at CB1-receptor. CHC bound both receptors at submicromolar ranges and was an agonist for these receptors. 2-HECBDV was an agonist at CB2-receptor and an antagonist at the CB1-receptor despite its modest affinity at this receptor (micromolar range). NMSC inhibited NF-κB and NFAT activity, and 2-HEC, 2-HPC, and GCBD dose-dependently inhibited PMA/IO-stimulated NFAT activation. CHC and HC dose-dependently reduced IL-1β and CCL2 messenger RNA (mRNA) expression. NMSC inhibited IL-1β, CCL2, and TNF-α at lower doses. At higher doses, it induced a pronounced increase in IL-6 mRNA. 2-HEC, 2-HPC, and GCBD dose-dependently inhibited LPS-induced IL-1β, TNF-α, and IL-6 synthesis. NMSC further increased LPS-stimulated IL-1β release but inhibited IL-8, TNF-α, and PGE_2_.

**Conclusion:** The CBD- and CBDV-derivatives studied are suitable for targeting CB-receptors. Some may be used as selective CB2 agonists. The length of the aliphatic rest at R^2^ of CBD (pentyl) and CBDV (propyl) did not correlate with the binding affinity. Higher polarity at R^1^ appeared to favor the agonistic activity at CB2-receptors.

## Introduction

The discovery of the endogenous cannabinoid (CB) system with its functional importance for the regulation and modulation of the immune and nervous system (Tanasescu and Constantinescu 2010; Booz, 2011) has led to a growing interest in natural and synthetic CBs. Since they have a very broad range of medically exploitable properties including antiemetic, appetite-enhancing, analgesic, antiphlogistic, muscle relaxing, sedative, anxiolytic, anti-depressive, antipsychotic, anti-oxidative, and neuroprotective effects, they are promising for the treatment of various indications (Massi et al. 2004; Pertwee, 2007; Booz 2011; Grotenhermen and Müller-Vahl 2012; Notcutt et al. 2012; Englund et al. 2013). These include side effects of chemotherapy, anorexia, pain, epilepsy, anxiety, multiple sclerosis, and Parkinson’s disease, which have been confirmed in numerous clinical trials (Grotenhermen and Müller-Vahl 2012; Hill et al. 2017). In the USA, the Food and Drug Administration (FDA) has approved cannabidiol (CBD) as an orphan drug for glioblastoma multiforme (a malignant primary brain tumor), and it has received the final approval for the treatment of severe epilepsy, namely, Lennox-Gastaut and Dravet syndromes.

The cost of CBs is high, because of their limited availability, extensive manufacturing costs, and regulatory limitations due to drug laws. In addition, for CBs to be used as effective medicine, it is important to have a reliable supply of the drug substances, as well as consistent quality and safety. Based on the work of Petrzilka et al. (1969) and Crombie et al. (1977), it is possible to produce CBD, dronabinol [(-)-*trans*-Δ9-THC], and cannabidivarin (CBDV) efficiently by continuous synthesis (Koch et al. 2013; Koch and Götz 2015). In our previous work, we synthesized CBD and CBDV through continuous synthesis in three steps from olivetol-carboxylic acid methyl ester (OM) and (+)-*p*-mentha-2,8-dien-1-ol (MD) (yield of 41%) or methyl divarin-carboxyl ester (DM) and MD (yield of 30%), respectively. Under optimized conditions, the purity after crystallization was >99%. By modifying the side groups at position 6 (R^1^) and position 5 (R^2^) of the allyl benzene moiety, this synthesis route offers CBs with a CBD or CBDV scaffold (**Table 1**) (Koch et al. 2013; Koch and Götz 2015). We created nine new CBs shown in **Table 1**.

**Table 1:** Newly synthesized cannabinoids.

R^2^	No.	R^1^	Abbreviation (log*P*)	Structure
-pentyl (CBD-derivative)	(**1**)	–CH_2_–CH_2_–OH	2-HEC (5.21)	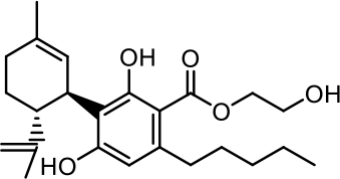
		2-Hydroxyethyl-		
	(**2**)	–CH_2_–CH(OH)–CH_2_–CH_2_–CH_3_	2-HPC (6.43)	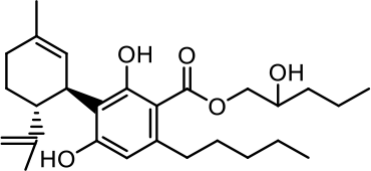
		2-Hydroxypentyl-		
	(**3**)	–CH_2_–CH(OH)–CH_2_–OH	GCBD (4.68)	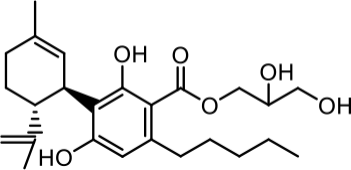
		2,3-Dihydroxypropyl-		
	(**4**)	–C_6_H_11_	CHC (7.81)	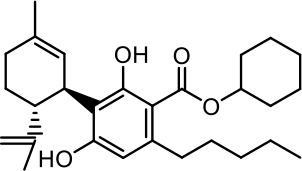
		Cyclohexyl-		
	(**5**)	–CH_2_–CH_2_–CH_2_–CH_2_–CH_2_–CH_2_	HC (7.28)	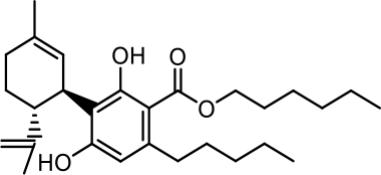
		*n*-Hexyl-		
	(**6**)	–CH_2_–CH_2_–NH–SO_2_–CH_3_	NMSC (3.84)	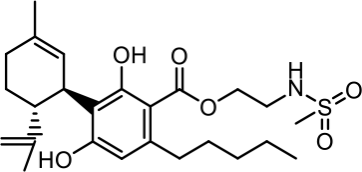
		2-(Methylsulfonamido)ethyl-		
-propyl (CBDV-derivative)	(**7**)	–CH_2_–CH_2_–OH	2-HECBDV (4.38)	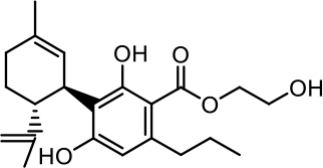
		2-Hydroxyethyl-		
	(**8**)	–C_6_H_11_	CHCBDV (6.44)	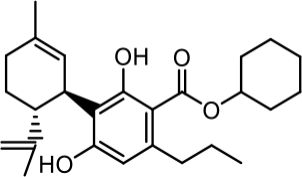
		Cyclohexyl-		
	(**9**)	–CH_2_–CH_2_–CH_2_–CH_2_–CH_2_–CH_2_	HCBDV (6.97)	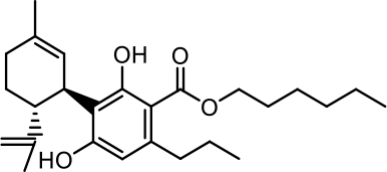
		*n*-Hexyl-		

Using in vitro and ex vivo cell models, we tested the binding and intrinsic effects at CB1- and CB2-receptors of the new CBs, as well as their effects on inflammatory signaling cascades. The CBD- and CBDV-derivatives studied here are suitable for targeting CB-receptors. In addition, some of the derivatives are promising as selective CB2 agonists.

## Material and Methods

### Synthesis

CBD- and CBDV-derivatives were synthesized by Friedel–Crafts alkylation based on the methods described by Koch et al. (2013) and Koch and Götz (2015) (Symbiosis Laboratories, Holzminden). Through Lewis acid catalysis, OMs and MD reacted in a continuous flow to form (-)-*trans*-CBD-carboxylic esters. CBDV-derivatives were accordingly synthesized through the reaction of divarin-carboxylic acid methyl esters with MD.

Six CBD-derivatives and three CBDV-derivatives have been synthesized: 2-hydroxyethyl cannabidiolate [2-HEC (**1**)], 2-hydroxypentyl cannabidiolate [2-HPC (**2**)], 2,3-dihydroxypropyl cannabidiolate [GCBD (**3**)], cyclohexyl cannabidiolate [CHC (**4**)], *n*-hexyl-cannabidiolate [HC (**5**)], 2-(methylsulfonamido)ethyl cannabidiolate [NMSC (**6**)], 2-hydroxyethyl cannabidivarinolate [2-HECBDV (**7**)], cyclohexyl cannabidivarinolate [CHCBDV (**8**)] and *n*-hexyl cannabidivarinolate [HCBDV (**9**)].

For the experiments, the stock solutions of the compounds were prepared in DMSO at 100 µmol/L and then diluted. The final DMSO concentration never exceeded 0.1% (*v*/*v*).

### Cell Culture

All cell lines were obtained from the American Type Culture Collection (ATCC, Manassas) and cultured according to standard procedures. Immortalized Chinese hamster ovary (CHO) cells and RAW264.7 macrophages were grown in Dulbecco’s modified Eagle’s medium (DMEM) containing 10% fetal bovine serum (FBS), 2 mmol -glutamine and 1% (*v*/*v*) penicillin/streptomycin (in sterile 0.9% NaCl aq, Sigma-Aldrich, St. Louis). Jurkat T-cells were cultured in RPMI-1640 medium (Sigma-Aldrich, St. Louis) with 10% FBS, 2 mmol -glutamine, 1 mmol HEPES (Life Technologies, Carlsbad), and 1% (*v*/*v*) penicillin/streptomycin (in sterile 0.9% NaCl aq, Sigma-Aldrich, St. Louis). Cells were maintained at 37°C in a humidified atmosphere containing 5% CO_2_.

Human peripheral blood monocytes were prepared from healthy donors who provided written informed consent at the local blood bank (University Hospital of Freiburg, Germany). Mononuclear cells were isolated by density gradient centrifugation according to standard protocol (English and Andersen, 1974) using lymphocyte separation medium, (PAN Biotech, Aidenbach). Cells were resuspended in RPMI-1640 medium (Invitrogen/Thermo Fisher Scientific, Karlsruhe) containing 10% human serum (Hexacell, Berlin). About 5 × 10^5^ cells per milliliter were seeded in 24-well plates and incubated at 37°C and 5% CO_2_. The medium and the non-adherent cells were removed by washing with PBS and fresh RPMI medium, and 1% human serum was added to each well.

### Binding Affinity

The derivatives were analyzed by competition studies (in a range of concentrations between 10^-12^ and 10^-4^ mol) that allow determining their affinity (K_i_ values) for both receptors against a classical CB ligand [^3^H]-CP55940 (164.5 Ci/mmol, Perkin Elmer, Boston). The competition studies were carried out with commercially available membranes prepared from CB1- or CB2-receptor-stably transfected HEK-293 cells (RBHCB1M400UA and RBXCB2M400UA; Perkin-Elmer Life and Analytical Sciences, Boston) following procedures previously published (Gómez-Cañas et al. 2016). Briefly, membranes were added in an assay buffer [for CB1: 50 mmol Tris-Cl, 5 mmol MgCl_2_·H_2_O, 2.5 mmol EDTA, 0.5 mg/ml bovine serum albumin, pH 7.4; or for CB2: 50 mmol Tris-Cl, 5 mmol MgCl_2_·H_2_O, 2.5 mmol ethylene glycol-bis(β-aminoethyl ether)-*N*,*N*,*N*′,*N*′-tetraacetic acid (EGTA), 1 mg/ml bovine serum albumin, pH 7.5) at a final concentration of 8 and 4 µg per well for CB1- and for CB2-receptors, respectively. The radioligand was used at 0.4 nmol for CB1-receptors or 0.53 nmol for CB2-receptors, always in a final volume of 200 µl for both receptors. The membranes were incubated for 90 min at 30°C with the radioligand and different concentrations of the different derivatives. Non-specific binding was determined with non-radiolabeled WIN55,212-2 (Sigma Aldrich, Madrid, 10 µmol) in the presence of radioligand. One hundred percent binding of the [^3^H]-CP55,940 was determined by incubation of the membranes with radioligand in the absence of any test compound. After incubation, free radioligand was separated from bound radioligand, by filtration in GF/C filters, previously treated with a 0.05% (*v*/*v*) polyethylenimine solution. Then, filters were washed nine times with a cold assay buffer, using the Harvester filtermate (Perkin Elmer, Boston). Radioactivity was measured using a liquid scintillation spectrometer (MicroBeta TriLux 1450 LSC & Luminescence Counter, Perkin Elmer, Boston). Data were expressed as percentage of [^3^H]-CP55940 binding and were analyzed, by using GraphPad Prism version 5 (GraphPad Software Inc., San Diego), for the calculation of K_i_ values for each receptor. They were expressed as mean ± SEM of at least three experiments performed in triplicate for each point.

### Signal Transduction

#### Signaling *via* the CB1- or CB2-Receptors

If the test compounds demonstrated an affinity for CB-receptors, the signaling profile of the compounds was examined in CHO cells transfected with CB1- and CB2-receptors in a cyclic adenosine monophosphate (cAMP)-dependent luciferase assay (Calandra et al. 1999; Sung et al. 2009). Briefly, CHO cells were stably transfected with CB1- and CB2-receptor complementary DNAs (cDNAs) (kindly provided by IMIBIC). Around 10^5^ transfected cells per milliliter were seeded in 24-well plates and transiently transfected with the plasmid CRE-luc using Roti-Fect (Carl Roth, Karlsruhe) following the manufacturer’s instructions. The cells were harvested after 24 h. Two transfected cell types resulted: CHO-CB1-CRE-luc and CHO-CB2-CRE-luc.

To test for agonistic activity at the CB1-receptor, the CHO-CB1-CRE-luc cells were stimulated for 6 h with increasing concentrations of the test compounds (Bouaboula et al. 1997). Forskolin (FSK), a strong CB-receptor-independent activator of adenylyl-cyclase was used at 10 µmol/L as a positive control. To investigate the antagonistic activity, the CHO-CB1-CRE-luc cells were pre-incubated for 15 min with the test compounds before stimulation for 6 h with 1 µmol/L WIN (Zheng et al. 2013). To investigate CB2-receptor agonistic activity, CHO-CB2-CRE-luc cells were pre-incubated for 15 min with increasing concentrations of the test substances and then for 6 h with 10 µmol/L FSK. WIN, a non-specific agonist at CB2-receptors, was used as a positive control. To confirm CB2-receptor agonistic activities of the test compounds, CHO-CB2-CRE-luc cells were pre-incubated with the specific CB2-receptor antagonist AM630 (1 µmol/L, 6-iodopravadoline; Cayman, distributed by Biozol, Hamburg) (Ross et al. 1999; Zheng et al. 2013) and then treated with increasing concentrations of the test substances.

### Activation of NF-κB and NFAT

Jurkat T-cells (10^6^ cells per milliliter) were transiently transfected with plasmids that contained an NF-κB-dependent (KBF-luc) or NFAT-dependent (NFAT-luc) promotors fused to the luciferase gene using Lipofectin (Life Technologies, Carlsbad) according to the manufacturer’s instructions (Yano et al. 1987; Durand et al. 1988). Twenty-four hours after transfection, cells were stimulated for 30 min with increasing doses of the test compounds or WIN as a control (Tocris, Bristol). Then PMA (50 ng/ml)—for NFAT-luc—plus ionomycin (0.5 µg/ml) (Sigma-Aldrich, St. Louis) was added, followed by a 6 h incubation period.

### Luciferase Activity Assay

After the stimulations and incubations, cells were lysed in a solution of 25 mmol Tris-phosphate (pH = 7.8), 8 mmol MgCl_2_, 1 mmol dithiothreitol (DTT), 1% Triton X-100, and 7% glycerol. The luciferase activity was measured with a Luciferase Assay Kit (Promega, Madison) according to the manufacturer’s instructions using an AutoLumat LB 9501 (Berthold Technologies, Bad Wildbad).

### IL-17-Induced Polarization

To assess the IL-17-induced polarization to pro-inflammatory M1 macrophages, RAW264.7 macrophages (10^6^ cells per Petri dish) were incubated for 18 h with or without the test compounds containing 0.1% FBS. Subsequently, they were stimulated for 24 h with recombinant murine IL-17 (50 ng/ml; R&D Systems, Minneapolis). The CB2-receptor agonist JWH-133 was used as a positive control. Then, the messenger RNA (mRNA) was extracted using RNeasy Mini Kits (Qiagen, Hilden), and its purity determined by UV measurements at 260 and 280 nm. The induction of IL-6, IL-1β, CC-chemokine ligand 2 (CCL2), and tumor necrosis factor (TNF)-α mRNA was assessed as markers of pro-inflammatory activity using quantitative real-time polymerase chain reactions (qPCRs). Primers (Eurofins Genomics, Ebersberg) were implemented at 5 µmol/L and are shown in **Table 2**.

**Table 2:** List of primers used.

Gene	Forward	Reverse
IL-6	5′-GAACAACGATGATGCACTTGC-3′	5′-TCCAGGTAGCTATGGTACTCC-3′
IL-1β	5′-CTCCACCTCAATGGACAGAA-3′	5′-GCCGTCTTTCATTACACAGG-3′
CCL2	5′-GGGCCTGCTGTTCACAGTT-3′	5′-CCAGCCTACTCATTGGGAT-3′
TNF-α	5′-CTACTCCCAGGTTCTCTTCAA-3′	5′-GCAGAGAGGAGGTTGACTTTC-3′
GAPDH	5′-TGGCAAAGTGGAGATTGTTGCC-3′	5′-AAGATGGTGATGGGCTTCCCG-3′

CCL2, CC-chemokine ligand 2; GAPDH, glyceraldehyde 3-phosphate dehydrogenase; IL, interleukin; TNF-*α*, tumor necrosis factor alpha.

mRNA reverse transcription in cDNA was performed with iQ™ SYBR Green Supermix and the CFX96 real-time PCR system (Bio-Rad, Hercules). The amplification consisted of a 5 min denaturation at 95°C followed by 40 cycles each of 30 s at 95°C and 30 s at 55°C (annealing). Elongation for 30 s at 72°C came next, followed by a cycle for 10 s at 83°C and finally a cycle for 1 min at 72°C. Calculations were made according to the Livak method by quantifying glyceraldehyde 3-phosphate dehydrogenase (GAPDH) as a reference (Schmittgen and Livak 2008). The results are given as multiples of the reference gene (2^ΔΔCt^).

### Anti-Inflammatory Effect on Human Monocytes

To test the anti-inflammatory effect of the test compounds, monocytes were incubated for 30 min with increasing doses of the test compounds. Then lipopolysaccharide (LPS) (10 ng/ml; *Escherichia coli*, Sigma-Aldrich, St. Louis) was added followed by a 24 h incubation period. The supernatants were removed and the levels of IL-1β, IL-6, IL-8, TNF-α, and prostaglandin E2 (PGE_2_) determined by enzyme-linked immunosorbent assay (ELISA) performed according to the manufacturer’s instructions (IL-1β from Hiss, Freiburg; IL-6, IL-8, and TNF-α from ImmunoTools, Friesoythe; and PGE_2_ from Cayman, distributed by Biozol, Hamburg). Stimulation with LPS alone was considered 100%.

#### Cytotoxicity Assays

Cytotoxicity tests were done on CHO cells, Jurkat T-cells, or RAW264.7 macrophages using the MTT assay (Sigma-Aldrich, St. Louis) and on primary human monocytes with the alamarBlue assay (Thermo Fisher Scientific, Karlsruhe).

### Statistical Analysis

At least three independent experiments were done in triplicate, and the results are given as mean ± SEM. Comparisons were performed using paired *t*-tests. Due to the explorative nature of the efficacy tests, a significance level of *α* = 10% was defined. For the same reason, error corrections for multiple tests were not done. Trend tests were performed using the Jonckheere–Terpstra test. All analyses were done with the statistics software R (version 3.4.4, www.r-project.org).

## Results

### Binding Affinity of CBs

The test compounds bound specifically to the CB-receptors at nanomolar or submicromolar ranges and, thus, physiologic concentrations, with the strongest affinity at the CB2-receptor for CHCBDV (**8**) and HCBDV (**9**) (K_i_ < 10 nmol/L) and the lowest for CHC (**4**) (K_i_ = 510 nmol/L). With the exception of NMSC (**6**), CHCBDV (**8**), and HCBDV (**9**) (highlighted in gray), the derivatives had a weak binding affinity for CB1-receptors (>500 µmol/L), having certain selectivity for CB2-receptors (**Table 3**). CHCBDV (**8**) and HCBDV (**9**) demonstrated strong binding to both receptors, whereas NMSC (**6**) was capable of binding to both receptors too, but with higher preference for the CB2-receptor (22.5-fold higher). The CBs are listed in **Table 3** in the order of decreasing selectivity for CB2-receptors.

**Table 3:** Inhibition constants (K_i_) and selectivity of test compounds for CB1- and CB2-receptors in the order of their selectivity for CB2-receptors. K_i_ values are expressed as the mean ± SEM of *n* = 3 independent experiments.

Test compound	K_i_ CB1 (nmol/L)	K_i_ CB2 (nmol/L)	Selectivity CB2/CB1
HC (**5**)	2,500 ± 900	67 ± 4	37.3
2-HECBDV (**7**)	5,649.3 ± 3,896.2	168.2 ± 39.4	33.6
NMSC (**6**)	270 ± 40	12 ± 1	22.5
2-HEC (**1**)	3,923 ± 1,547	374.5 ± 47.7	10.5
2-HPC (**2**	538.2 ± 53.9	66.7 ± 13.1	8.1
GCBD (**3**)	2,174 ± 1,149	277.1 ± 78.7	7.8
CHCBDV (**8**)	13.2 ± 0.9	4.62 ± 0.5	2.9
CHC (**4**)	870 ± 100	510 ± 290	1.7
HCBDV (**9**)	8.3 ± 0.69	9.9 ± 2.5	0.8
WIN55,212-2 (reference compound)	28.8 ± 41	3.7 ± 1	-

CB, cannabinoid; CHC, cyclohexyl cannabidiolate; CHCBDV, cyclohexyl cannabidivarinolate; GCBD, 2,3-dihydroxypropyl cannabidiolate; HC, n-hexyl-cannabidiolate; HCBDV, n-hexyl cannabidivarinolate; NMSC, 2-(methylsulfonamido)ethyl cannabidiolate; 2-HEC, 2-hydroxyethyl cannabidiolate; 2-HECBDV, 2-hydroxyethyl cannabidivarinolate; 2-HPC, 2-hydroxypentyl cannabidiolate.

### Intrinsic Activity of the CB Derivatives

#### CB-Receptor-Mediated Signal Transduction

**Figure 1:**
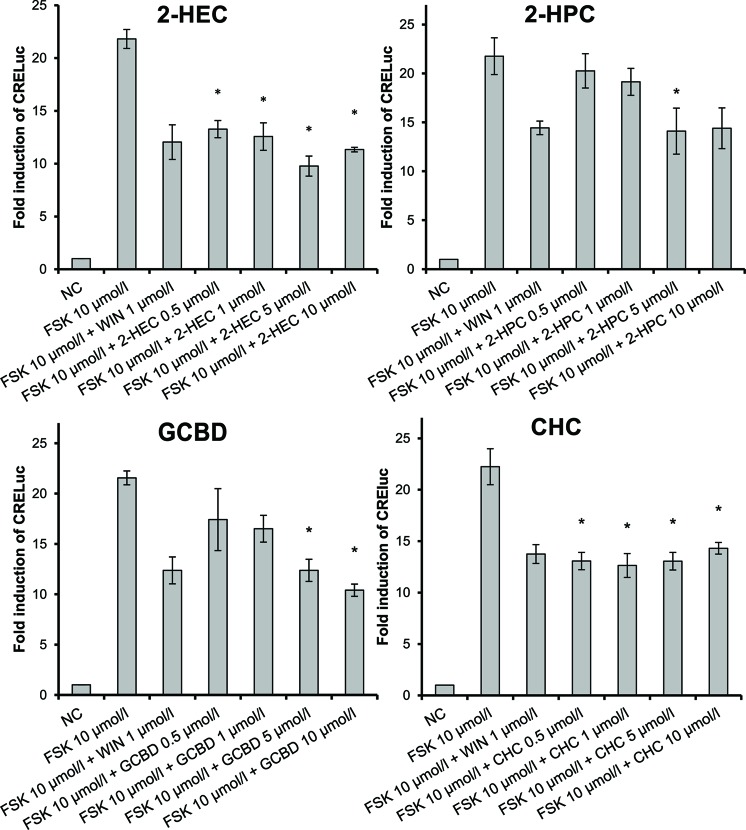
CB2-receptor-mediated agonistic effects given as fold induction of CRE-luc; CB, cannabinoid; NC, negative control; FSK, forskolin; WIN, WIN 55212-2; error bars represent SEM, *p < 0.1.

To assess whether the test compounds were able to activate CB-receptors, signaling via the CB-receptors was tested using transfected CHO cells (CHO-CB1-CRE-luc and CHO-CB2-CRE-luc). As determined with the MTT assay, the test compound doses used in the assay ranged from 0.5 to 10 µmol/L. The CBD-derivatives 2-HEC (**1**), 2-HPC (**2**), GCBD (**3**), CHC (**4**), and NMSC (**6**) activated the CB2-receptor and acted as agonists (**Figures 1** and **4**). 2-HEC (**1**) and CHC (**4**) demonstrated effects that were as strong as the same dose of the positive control (1 µmol/L WIN). 2-HPC (**2**), GCBD (**3**), and 2-HECBDV (**7**) showed dose-dependent agonistic effects on CB2-receptors (trend test *p* = 0.026, 0.01, and 0.026, respectively). 2-HEC (**1**) displayed dose dependency at concentrations between 0.5 and 5 µmol/L. Only HC (**5**) did not activate the CB2-receptor (data not shown), despite the K_i_ values for this receptor indicating that it could be active.

We checked the agonistic effect of the test compounds; they were tested against the inverse agonist AM630. The agonism was confirmed, and none of the substances showed a tendency toward CB2-receptor antagonism (data not shown).

**Figure 2:**
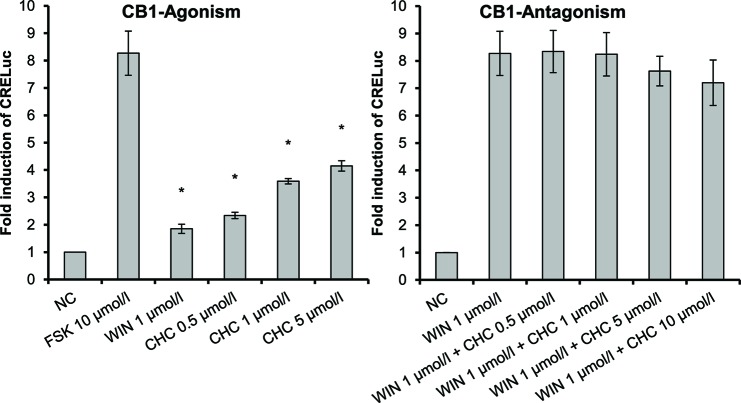
CB1-receptor-mediated agonistic effect of CHC (**4**) given as fold induction of CRE-luc; CB, cannabinoid; CHC, cyclohexyl cannabidiolate, NC; negative control, FSK: forskolin; WIN, WIN 55212-2; error bars represent SEM, *p < 0.1.

CHC (**4**) was the only compound to activate CB1-receptors (**Figure 2**). Tests of the antagonistic effect against this receptor were negative.

**Figure 3:**
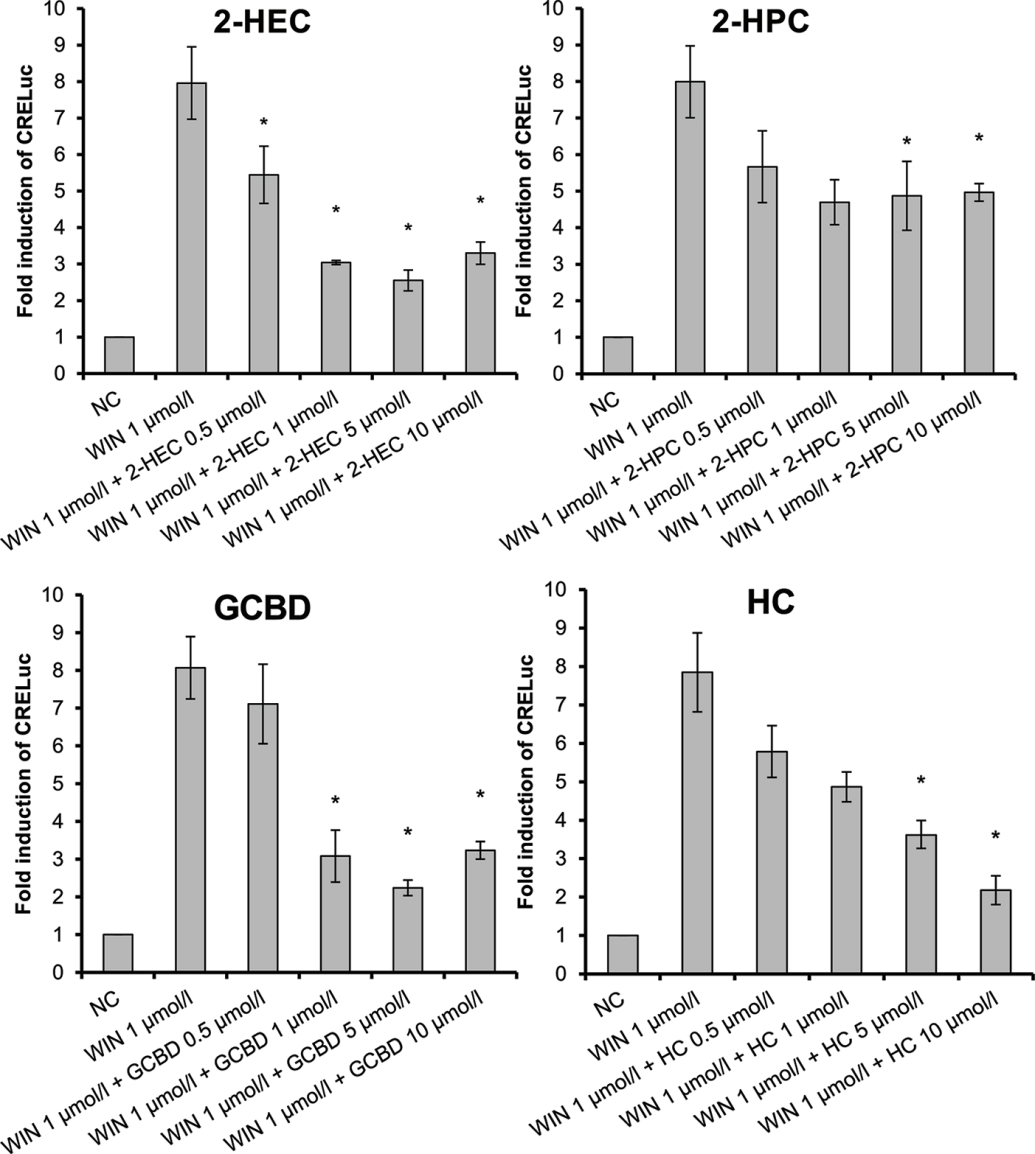
CB1-receptor-mediated antagonistic effect of 2-HEC (**1**), 2-HPC (**2**), GCBD (**3**), and HC (**5**) given as fold induction of CRE-luc; GCBD, 2,3-dihydroxypropyl cannabidiolate, HC, *n*-hexyl-cannabidiolate, NC, negative control; 2-HEC, 2-hydroxyethyl cannabidiolate; 2-HPC, 2-hydroxypentyl cannabidiolate; WIN, WIN 55212-2; error bars represent SEM, *p < 0.1.

**Figure 4:**
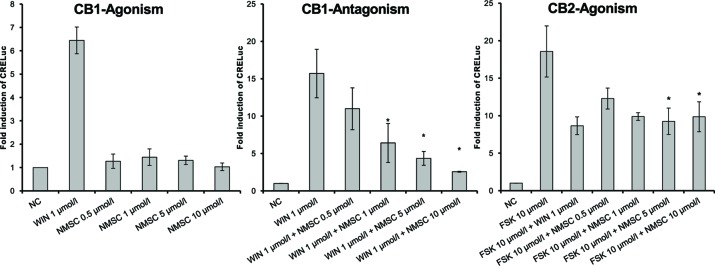
CB1- and CB2-receptor-mediated effects of NMSC (**6**) given as fold induction of CRE-luc; CB, cannabinoid; FSK, forskolin; NC, negative control; NMSC, 2-(methylsulfonamido)ethyl cannabidiolate, WIN, WIN 55212-2; error bars represent SEM, *p < 0.1.

2-HEC (**1**), 2-HPC (**2**), GCBD (**3**), and HC (**5**) acted as antagonists at CB1-receptors (**Figure 3**) (*p* < 0.1). HC (**5**) and NMSC (**6**) demonstrated dose-dependent activity (**Figures 3** and **4**; *p* < 0.001 and *p* = 0.017, respectively).

Interestingly, NMSC (**6**) presented a high affinity for both receptors. The compound was an antagonist at CB1-receptors and an agonist at CB2-receptors (**Figure 4**). NMSC (**6**) had a dose-dependent effect (trend test *p* = 0.017) that, at the same concentration as WIN (1 µmol/L), was strongly CB1 antagonistic and similarly strongly agonistic at CB2.

**Figure 5:**
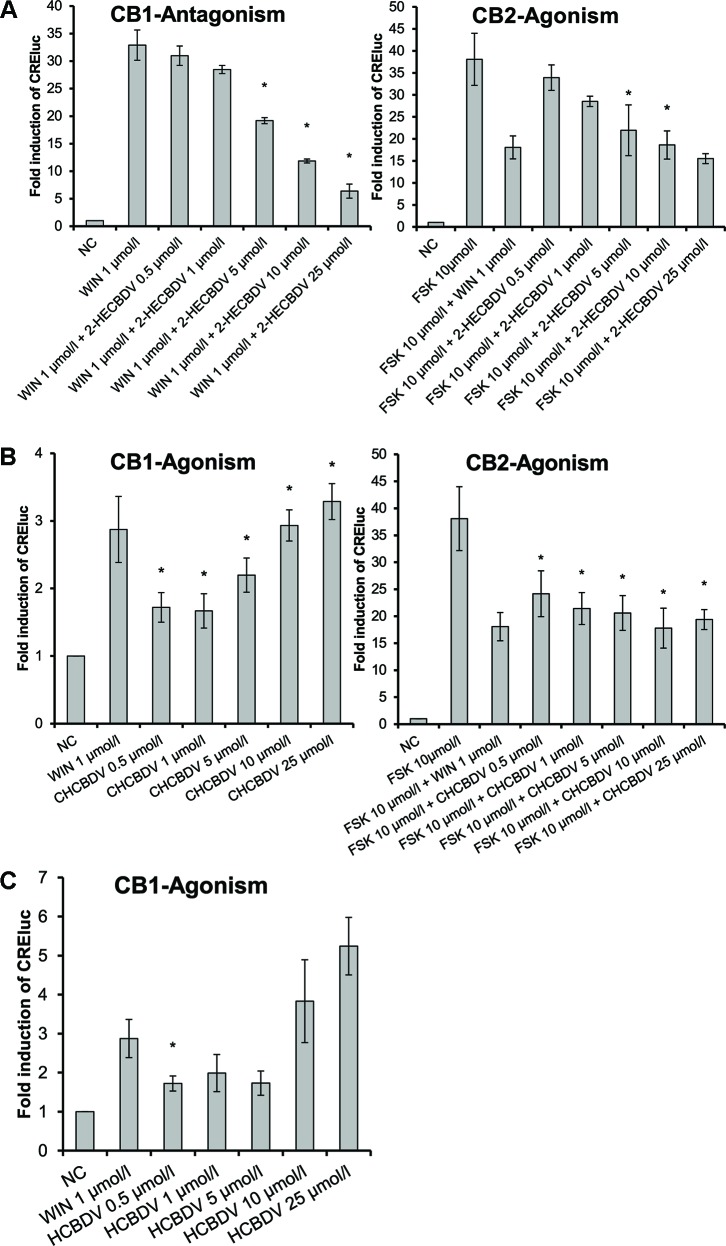
CB-receptor-mediated effect of CBDV-derivatives. The following compounds were tested: **(A)** 2-HECBDV (**7**), **(B)** CHCBDV (**8**), and **(C)** HCBDV (**9**). Given are fold induction of CRE-luc; CB, cannabinoid; CBDV, cannabidivarin; CHCBDV, cyclohexyl cannabidivarinolate; HCBDV, *n*-hexyl cannabidivarinolate; NC, negative control; WIN, WIN 55212-2; 2-HECBDV, 2-hydroxyethyl cannabidivarinolate; error bars represent SEM, *n* = 4 independent experiments, *p < 0.1.

The three CBDV-derivatives studied demonstrated heterogeneous effects on the CB-receptors. Similar to the CBD-derivatives, 2-HECBDV (**7**) was an antagonist at CB1-receptors and an agonist at CB2-receptors (**Figure 5A**). The effect was dose dependent ranging from 0.5 to 25 µmol/L (trend tests: *p* ≤ 0.001 and *p* = 0.026, respectively). CHCBDV (**8**) demonstrated a non-selective agonism at both CB1- and CB2-receptors (**Figure 5B**). The effect on CB1 was dose dependent (*p* = 0.001). HCBDV (**9**) displayed agonistic effects on CB1-receptors and had no effect on CB2 (**Figure 5C**). The trend test showed a dose dependency (*p* = 0.025).

**Table 4** summarizes the effects of the tested compounds on the CB-receptors. All CBD-derivatives except HC (**5**) had an agonistic effect on CB2-receptors. At the same time, with the exception of CHC (**4**), they demonstrated an antagonistic effect on CB1.

**Table 4:** Effect of cannabidiol (CBD)- and cannabidivarin (CBDV)-derivatives on cannabinoid CB1-and CB2-receptors (agonistic activity highlighted in gray).

Test compound	Effect on CB1	Effect on CB2
2-HEC (**1**)	Antagonist	Agonist
2-HPC (**2**)	Antagonist	Agonist
GCBD (**3**)	Antagonist	Agonist
CHC (**4**)	Agonist	Agonist
HC (**5**)	Antagonist	No activity
NMSC (**6**)	Antagonist	Agonist
2-HECBDV (**7**)	Antagonist	Agonist
CHCBDV (**8**)	Agonist	Agonist
HCBDV (**9**)	Agonist	No activity

CB, cannabinoid; CHC, cyclohexyl cannabidiolate; CHCBDV, cyclohexyl cannabidivarinolate; GCBD, 2,3-dihydroxypropyl cannabidiolate; HC, n-hexyl-cannabidiolate; HCBDV, n-hexyl cannabidivarinolate; NMSC, 2-(methylsulfonamido)ethyl cannabidiolate; 2-HEC, 2-hydroxyethyl cannabidiolate; 2-HECBDV, 2-hydroxyethyl cannabidivarinolate; 2-HPC, 2-hydroxypentyl cannabidiolate.

The basic structure of CBD-derivatives was more likely to demonstrate agonistic effects than CBDV-derivatives (five of six vs. two of three derivatives, respectively). Therefore, due to the limited conclusions that can be drawn from the small number of tested substances, subsequent testing was done with the six CBD-derivatives.

### Effects of CBD-Derivatives on CB2-Receptor-Expressing Immune Cells

**Figure 6:**
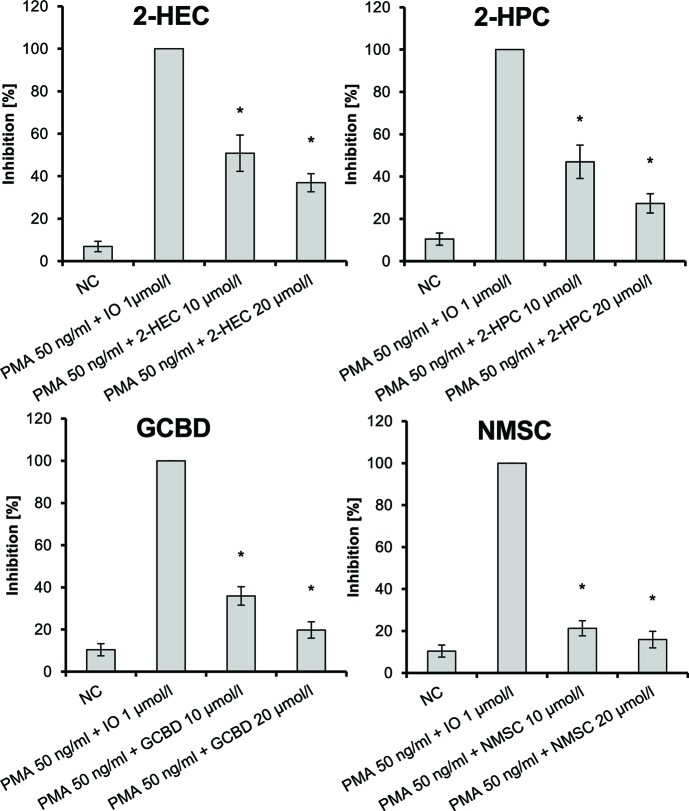
Inhibition of PMA/IO-induced NFAT activation in Jurkat T-cells given as the percent reduced expression of NFAT-luc; IO, ionomycin; NC, negative control; NFAT, nuclear factor of activated T-cells; PMA, phorbol-12-myristate-13-acetate; *n* = 3 independent experiments; error bars represent SEM, *p < 0.1.

CB2-receptor agonists inhibit signaling via NF-κB or NFAT in CB2-receptor-expressing Jurkat T-cells. As the CBD-derivatives proved to be agonists at CB2, it was checked whether this pathway can be inhibited in Jurkat cells. CBD-derivatives were used at a dose of 0.5–25 µmol/L as determined in an MTT assay. 2-HEC (**1**), 2-HPC (**2**), GCBD (**3**), and NMSC (**6**) significantly inhibited PMA/IO-induced NFAT activation of Jurkat T-cells (**Figure 6**) in a dose-dependent fashion (trend tests: *p* = 0.038, *p* = 0.005, *p* = 0.006, and *p* = 0.06, respectively).

**Figure 7:**
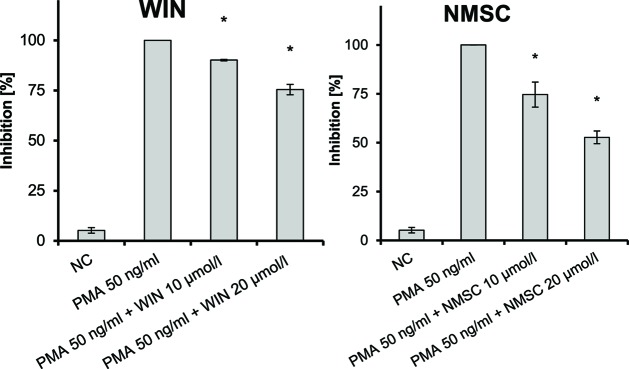
Inhibition of PMA-induced NF-κB activation in Jurkat T-cells given as the percent reduced expression of NF-κB-luc; NC, negative control; NF-κB, nuclear factor κB; PMA, phorbol-12-myristate-13-acetate; *n* = 3 independent experiments; error bars represent SEM, *p < 0.1.

NMSC (**6**) also inhibited the PMA-induced NF-κB activation in Jurkat T-cells in a dose-dependent manner (**Figure 7**; *p* = 0.003) similar to WIN. The inhibition on NF-kB activation was only marginal with HC (**5**), while no inhibition was seen on the activation of NFAT (data not shown). CHC (**4**) did not inhibit PMA-mediated activation of either NFAT or NF-κB.

### Effect On IL-17-Mediated Polarization To Pro-Inflammatory M1 Macrophages

We studied whether the test compounds could inhibit the IL-17-mediated polarization to pro-inflammatory M1 macrophages using RAW264.7 macrophages. CHC (**4**), HC (**5**), and NMSC (**6**) were tested on macrophages at doses ranging from 0.5 to 10 µmol/L as determined in an MTT assay. 2-HEC (**1**), 2-HPC (**2**), and GCBD (**3**) had a negative effect on cell viability at concentrations of 5 and 10 µmol/L. Therefore, they were excluded from examination on RAW264.7 cells, because the toxicity was expected to influence the results of the functional experiments.

**Figure 8:**
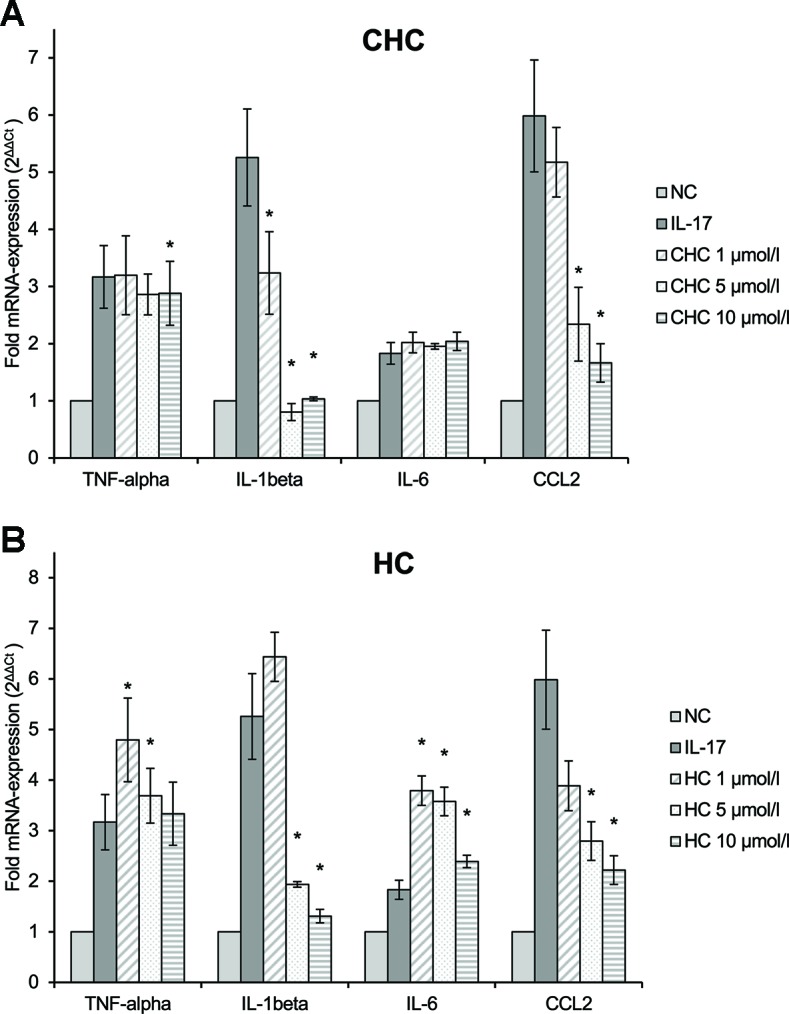
Effect of CHC **(A)** and HC **(B)** on the IL-17-stimulated mRNA expression of TNF-α, IL-1β, IL-6, and CCL2 in RAW264.7 macrophages given as multiples of the reference gene expression. CCL2, CC-chemokine ligand 2; CHC, cyclohexyl cannabidiolate; HC, *n*-hexyl-cannabidiolate; IL, interleukin; mRNA, messenger RNA; NC, negative control; TNF, tumor necrosis factor; *n* = 3 independent experiments; error bars represent SEM, *p < 0.1.

RAW264.7 macrophages that were pre-incubated with CHC (**4**) (**Figure 8A**) inhibited the M1 markers IL-1β and CCL2. The latter was reduced in a dose-dependent manner (*p* = 0.008). HC (**5**) (**Figure 8B**) also inhibited IL-1β and CCL2 mRNA expressions, both dose dependently (*p* = 0.002 and *p* = 0.013, respectively). Due to the low induction of IL-6 and TNF-α by IL-17, their inhibition was not detectable or so weak that definite conclusions cannot be made. It seems that HC had a stimulating effect at low doses. The trend test showed a dose-dependent reduction in IL-6 (*p* = 0.013).

**Figure 9:**
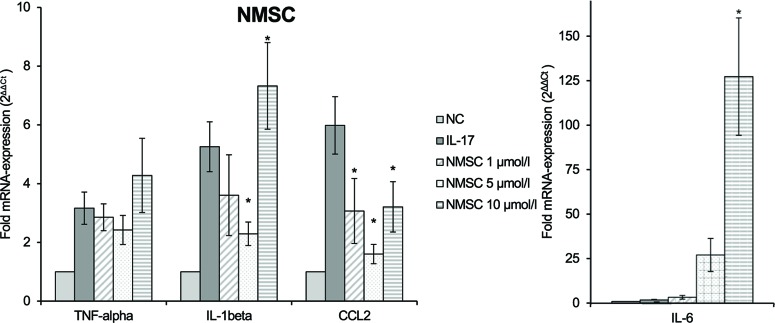
Effect of NMSC on the IL-17-stimulated mRNA expression of TNF-α, IL-1β, IL-6, and CCL2 in RAW264.7 macrophages given as multiples of the reference gene expression. CCL2, CC-chemokine ligand 2; IL, interleukin; mRNA, messenger RNA; NC, negative control, NMSC; 2-(methylsulfonamido)ethyl cannabidiolate; TNF, tumor necrosis factor; *n* = 3 independent experiments; error bars represent SEM, *p < 0.1.

In contrast, NMSC (**6**) inhibited TNF-α and IL-1β only at low doses, while it activated gene expression at high doses (**Figure 9**). CCL2 was reduced compared to the control at all concentrations. At the same time, NMSC (**6**) co-stimulated IL-6 in a dose-dependent manner (*p* = 0.002).

### Anti-Inflammatory Effects of the Test Compounds

The anti-inflammatory properties of the test compounds were studied in cultures of primary human monocytes treated with LPS (10 ng/ml). These unspecific mononuclear immune cells are involved in the first steps of inflammatory reactions. Cytotoxicity tests showed that cell viability was only slightly affected at concentrations of 0.1–10 µmol/L of the compounds. Therefore, a maximum of 10 µmol/L test compound was chosen with the exception of NMSC (**6**), which was used at a maximum of 2.5 µmol/L.

**Figure 10:**
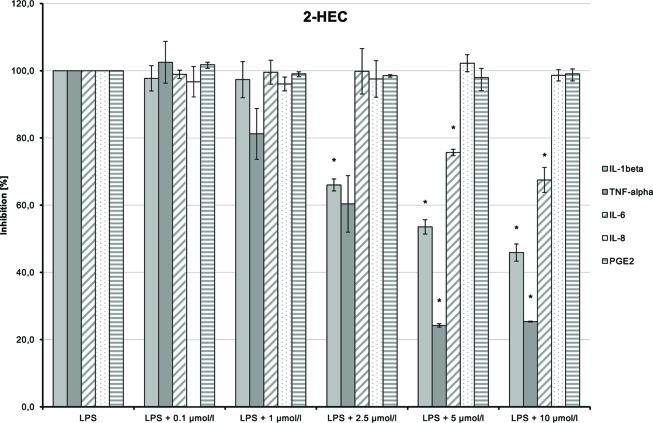
Anti-inflammatory effect of 2-hydroxyethyl cannabidiolate (2-HEC) on primary human monocytes; normalized to the induction by lipopolysaccharide (LPS) (without the addition of test compounds); *n* = 2 independent experiments; error bars show SEM, *p < 0.1.

**Figure 11:**
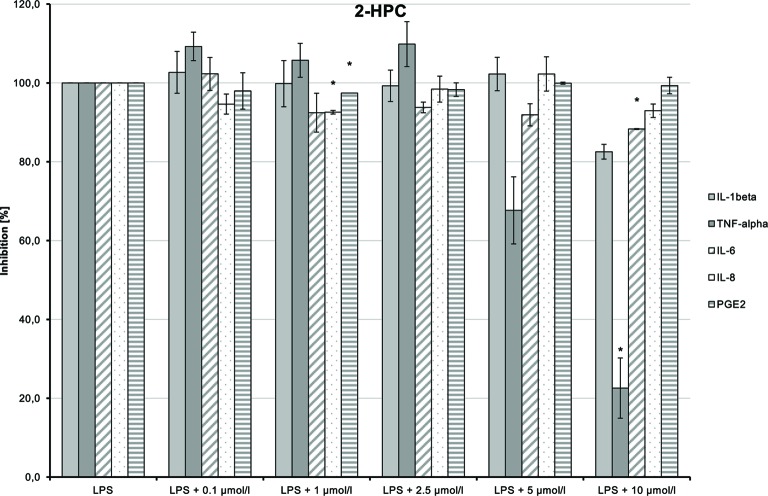
Anti-inflammatory effect of 2-hydroxypentyl cannabidiolate (2-HPC) on primary human monocytes; normalized to the induction by lipopolysaccharide (LPS) (without the addition of test compounds); *n* = 2 independent experiments; error bars show SEM, *p < 0.1.

**Figure 12:**
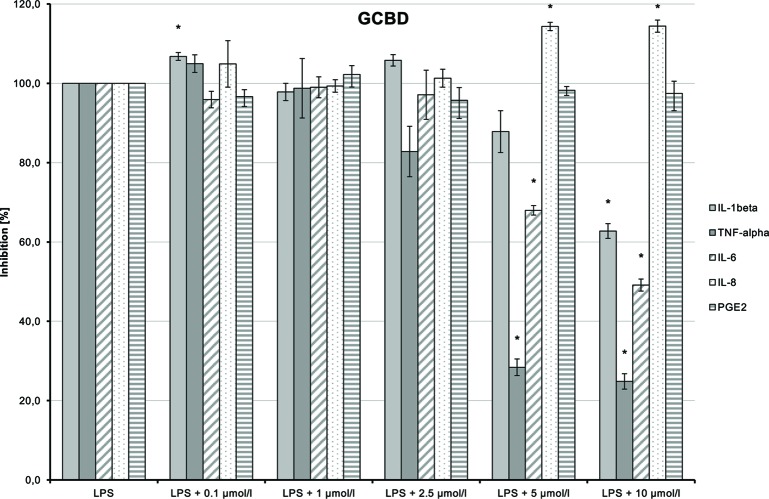
Anti-inflammatory effect of 2,3-dihydroxypropyl cannabidiolate (GCBD) on primary human monocytes; normalized to the induction by lipopolysaccharide (LPS) (without the addition of test compounds); *n* = 2 independent experiments; error bars show SEM, *p < 0.1.

LPS-induced IL-1β, TNF-α, and IL-6 releases were significantly inhibited in a dose-dependent manner by 2-HEC (**1**) (*p* = 0.003, *p* = 0.003, *p* = 0.049) and GCBD (**3**) (*p* = 0.04, *p* = 0.004, *p* < 0.1) (each respectively; **Figures 10**and**12**). The strongest IL-1β inhibition was seen for 2-HEC (**1**) (**Figure 10**). Only TNF-α and IL-6 releases were significantly inhibited by 2-HPC (**2**) (*p* = 0.017, *p* = 0.008; **Figure 11**).

LPS-stimulated IL-8 and PGE_2_ synthesis was inhibited at a dose of 1 µmol/L by 2-HPC (**2**) (**Figure 11**). GCBD (**3**)costimulated LPS-mediated release of IL-8 significantly at higher doses (**Figure 12**).

**Figure 13:**
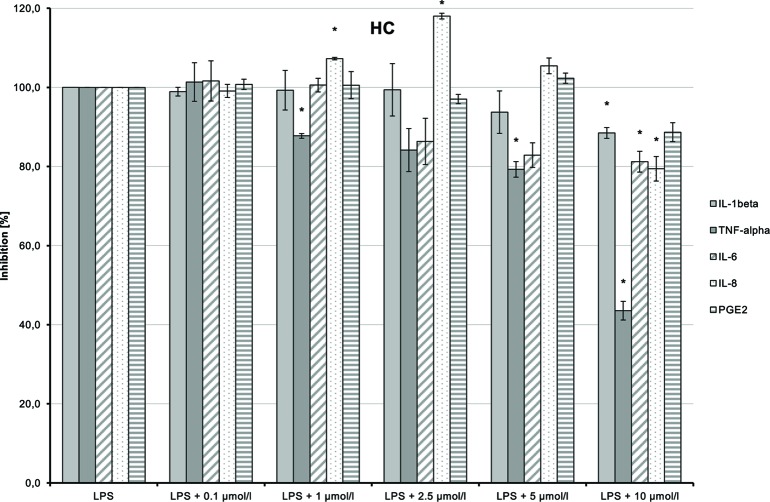
Anti-inflammatory effect of n-hexyl-cannabidiolate (HC) on primary human monocytes; normalized to the induction by lipopolysaccharide (LPS) (without the addition of test compounds); *n* = 2 independent experiments; error bars show SEM, *p < 0.1.

HC (**5**) dose-dependently inhibited LPS-induced production of TNF-α and IL-6 (**Figure 13**; *p* = 0.006 and *p* = 0.029, respectively). IL-1β and IL-8 syntheses stimulated by LPS were also inhibited at higher doses of the substance. The inhibition of all markers using the high dose of 10 µmol/L may possibly be a cytotoxic and thus unspecific effect.

**Figure 14:**
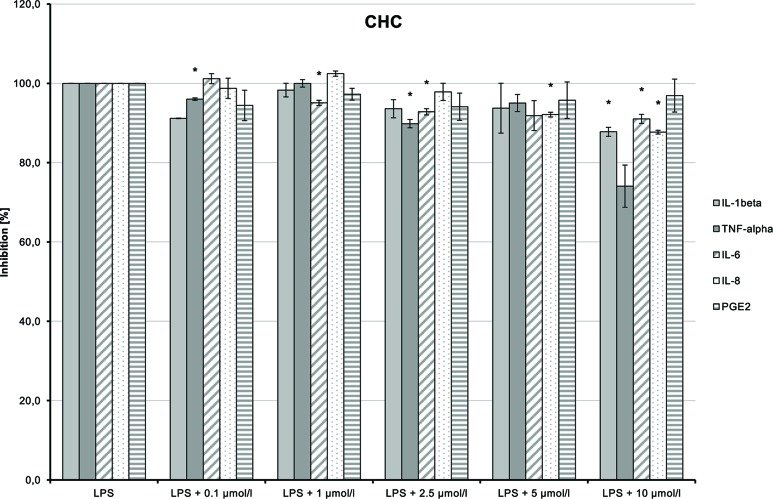
Anti-inflammatory effect of cyclohexyl cannabidiolate (CHC) on primary human monocytes; normalized to the induction by lipopolysaccharide (LPS) (without the addition of test compounds); *n* = 2 independent experiments; error bars show SEM, *p < 0.1.

CHC (**4**) had only very weak anti-inflammatory effects (**Figure 14**). LPS-induced IL-6 and IL-8 releases were moderately and dose-dependently inhibited (*p* = 0.008 and *p* = 0.016, respectively). TNF-α and IL-1β synthesis stimulated by LPS was also reduced, but the trend test indicated no dose-dependent effect.

**Figure 15:**
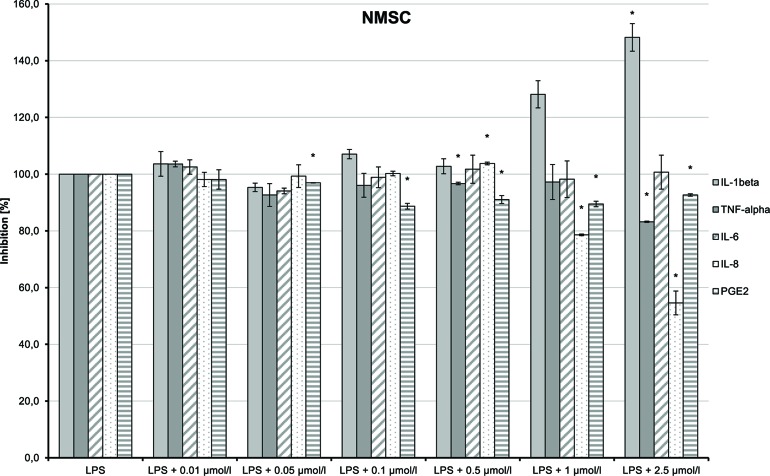
Pro-inflammatory and anti-inflammatory effects of 2-(methylsulfonamido)ethyl cannabidiolate (NMSC) on primary human monocytes; normalized to the induction by lipopolysaccharide (LPS) (without the addition of test compounds); *n* = 2 independent experiments; error bars show SEM, *p < 0.1.

NMSC (**6**) enhanced the LPS-induced synthesis of IL-1β, which suggests an immunity-enhancing effect of the compound (**Figure 15**). Concomitantly, LPS-induced releases of TNF-α, IL-8, and PGE_2_ were significantly reduced.

## Discussion

The novel CBD- and CBDV-derivatives created by continuous synthesis and by modifying the side groups can target CB-receptors, which is in contrast to the poor affinity for CB1-/CB2-receptors of their source compounds CBD and CBDV (Rosenthaler et al. 2014). Some of the substances had binding affinities to both receptors in a similar range as Δ9-THC and similar effects as WIN (Kapur et al. 2009; Pertwee et al. 2010).

The binding affinity of the test compounds did not allow us to correlate between the aliphatic side chain (pentyl for CBD-derivatives and propyl for CBDV-derivatives) at position 5 (**Table 1**) and the selectivity of the CBs. In addition, derivatives with long aliphatic side chains at the ester at position 6 [HC (**5**)] as well as the ones with polar side chains [2-HECBDV (**7**), NMSC (**6**), and 2-HEC (**1**)] can be selective for CB2-receptors.

Test compounds with a sterically rigid apolar rest [CHCBDV (**8**) and CHC (**4**)] as well as those with a less rigid one [HCBDV (**9**)] demonstrated moderate to high affinity for both CB-receptors. However, this cannot be considered a general rule, because HC (**5**)—a substance with an apolar rest—demonstrated a strong selectivity for CB2-receptors.

The CBD-derivatives (five of six) were more likely to have an agonistic effect on CB2-receptors than the CBDV compounds (two of three). However, the small number of tested substances limits this conclusion, and further studies are necessary.

As in the case of HC (**5**), strong binding affinity to the CB2-receptor alone was not sufficient to activate this receptor. Nevertheless, it exhibited anti-inflammatory effects as shown in RAW264.7 macrophages and primary human monocytes. This might be due to interaction with other receptors, metabolites, or through allosteric modulation.

It is interesting that NMSC (**6**) and 2-HECBDV (**7**), which had the lowest log*P* values (3.84 and 4.38, respectively) both had an agonistic effect on CB2-receptors and an antagonistic effect on CB1-receptors. This is possibly due to the higher polarity of the side chain at position 6.

Our results on the activation of the transcription factors NFAT and NF-κB in T-cells strongly indicate that the tested CB2-agonists are more likely to inhibit the NFAT signaling pathway than the NF-κB pathway. The agonistic effect of the CBD-derivatives was confirmed in CB2-receptor-expressing immune cells. In Jurkat T-cells, 2-HEC (**1**), 2-HPC (**2**), GCBD (**3**), and NMSC (**6**) inhibited PMA/IO-induced NFAT activation. NMSC (**6**) as the substance with the lowest log*P*-value also inhibited PMA-stimulated activation of NF-κB. The more apolar CBD-derivatives HC (**5**) and CHC (**4**) demonstrated only a marginal or no effect on both signaling pathways.

Activation of the CB2-receptor leads to potent anti-inflammatory properties (Klein, 2005; Cencioni et al., 2010; Guillot et al., 2014). As expected, the CB2-agonists CHC (**4**) and NMSC (**6**) inhibited the IL-17-mediated polarization of RAW264.7 macrophages to pro-inflammatory M1 macrophages. We found that CBD-derivatives with apolar rests, HC (**5**) and CHC (**4**), demonstrated a dose-dependent inhibition of mRNA expression of IL-1β and CCL2. Similar to the strongly polar NMSC (**6**), they also showed a weak inhibition of TNF-α expression. The more apolar substances were consistent in inhibiting M1 polarization while the sulfonamide led to an increase in inflammatory markers at higher doses, especially IL-6. This may correlate with the increased binding affinity of this compound that may create paradoxical effects at higher doses.

All six tested CBD-derivatives displayed inhibition of LPS-induced TNF-α induction in primary monocytes. In five of the CBDs, the reduction was dose dependent. A correlation of the effect and the compounds’ polarity or the large rest was not observed. The CBD basic structure seems to have an anti-inflammatory effect in primary human immune cells. The strongly polar sulfonamide side chain, however, enhanced LPS-induced IL-1β and IL-6 production.

Noteworthy is that higher polarity of the molecules, due to the side chain R^1^ [2-HECBDV (**7**), NMSC (**6**), GCBD (**3**), and 2-HEC (**1**)], seems to favor the agonistic activity at CB2-receptors. Nevertheless, molecules with lower polarity [CHC (**4**) and CHCBDV (**8**)] also showed agonistic activity at CB2.

To increase the significance of our results on structure–effect relationships, additional synthetic derivatives and their testing are necessary.

## Data Availability Statement

The datasets for this manuscript are not publicly available because of the tenure of intellectual property by Symrise AG. Requests to access the datasets should be directed to the correspondence author.

## Author Contributions

MG led and substantially contributed to the conception and design of the work and the acquisition, analysis, and interpretation of data for the work. UH supervised the doctoral thesis of MG and contributed to the analysis and interpretation of data for the work. JF-R, BF, OK, and EM contributed to the conception or design of the work or to the acquisition, analysis, or interpretation of data for the work. CN, JC, LG-T, MG-C and MP contributed to the acquisition, analysis, and interpretation of data for the work. AL contributed to the analysis of data for the work.The authors were involved in drafting or critically revising the work for intellectual content. The authors approved of the publication of the content and agreed to be accountable for all aspects of the work in ensuring that questions related to the accuracy or integrity of any part of the work are appropriately investigated and resolved.

## Funding

This work was funded by Symrise AG, Holzminden, Germany.

## Conflict of Interest

MG and OK are employees of Symrise AG, Holzminden. JF-R, LG-T, MG-C and MP are employees of the Universidad Complutense, Madrid, and performed screening experiments commissioned by Symrise AG. BF is an employee of Vivacell Biotechnology GmbH and performed screening experiments commissioned by Symrise AG: JC and CM are employee of Vivacell Biotechnology España and performed screening experiments commissioned by Symrise AG. EM is an employee of University of Córdoba, Córdoba, and performed screening experiments requisitioned by Symrise AG. AL is an employee of the Medical Biometry and Statistical Bioinformatics, Department of Medical Statistics, Georg-August-University of Göttingen and performed data analyses requisitioned by Symrise AG.

The remaining authors declare that the research was conducted in the absence of any commercial or financial relationships that could be construed as a potential conflict of interest.
